# Lactic Acid Bacteria-Fermentable Cereal- and Pseudocereal-Based Beverages

**DOI:** 10.3390/microorganisms9122532

**Published:** 2021-12-07

**Authors:** Małgorzata Ziarno, Patrycja Cichońska

**Affiliations:** Department of Food Technology and Assessment, Institute of Food Science, Warsaw University of Life Sciences-SGGW (WULS-SGGW), 02-787 Warsaw, Poland; patrycja_cichonska@sggw.edu.pl

**Keywords:** plant-based beverages, lactic acid fermentation, bioactive metabolites, B vitamins

## Abstract

Plant beverages are becoming more popular, and fermented cereal- or pseudocereal-based beverages are increasingly used as alternatives for fermented products made from cow milk. This review aimed to describe the basic components of cereal- or pseudocereal-based beverages and determine the feasibility of fermenting them with lactic acid bacteria (LAB) to obtain products with live and active LAB cells and increased dietary value. The technology used for obtaining cereal- or pseudocereal-based milk substitutes primarily involves the extraction of selected plant material, and the obtained beverages differ in their chemical composition and nutritional value (content of proteins, lipids, and carbohydrates, glycemic index, etc.) due to the chemical diversity of the cereal and pseudocereal raw materials and the operations used for their production. Beverages made from cereals or pseudocereals are an excellent matrix for the growth of LAB, and the lactic acid fermentation not only produces desirable changes in the flavor of fermented beverages and the biological availability of nutrients but also contributes to the formation of functional compounds (e.g., B vitamins).

## 1. Introduction

Milk obtained from other mammals is an indispensable part of the human diet. Lactase, an enzyme produced by our body, allows the absorption of nutrients from milk by catalyzing the hydrolysis of lactose, which is the predominant milk carbohydrate. The activity of lactase is highest in infants, but as the diet expands, the enzyme’s activity gradually reduces. This is the most common cause of food intolerance associated with the consumption of dairy products. People with insufficient lactase activity are forced to abstain from consuming cow milk and its products [1,2,3,4]. Diets that do not include some food products, such as milk and its products (e.g., hypoallergenic diet or vegan diet), have been increasingly adopted by people in recent times. Cow milk substitutes are gaining attention as people switch to a plant-based diet and as consumer awareness regarding food production, particularly protein sources, has been on the rise [5,6,7,8]. In addition, the plant diet has been on trend for several years, and people who are eager to follow the new trend in nutrition and seek milk alternatives or new flavors reach for vegetable beverages, which are often referred to as milk-like beverages. Plant beverages are becoming more popular, and researchers are currently focusing on improving the organoleptic features, texture, and stability of these types of beverages and sustainable nutrition [5,6,7,9,10,11,12].

Alternative products are available from the food industry for people who avoid milk and derivative products for various reasons. These substitutes are mainly beverages of plant origin, which to some extent replace the products obtained from cow milk. From a technological standpoint, cow milk substitutes are typically aquatic extracts of a crumbled plant material. In the production process, homogenization is mostly applied to improve fluid homogeneity, as well as thermal treatment, which aids in increasing the stability of microbiological products [13]. Plant-based substitutes are similar to cow milk in appearance but differ in nutrients as well as taste and smell. The nutrient value and taste of plant-derived products are determined by many factors, including the origin of the plant material used for their production, the processing method, and the substances added for enrichment.

The demand for alternative dairy products for the production of plant-based milk replacements has been met with vegetable raw materials. The commonly used plant sources can be divided into the following types [14,15]:

- Legumes (e.g., soy, with lupines, mung beans, adzuki beans)
- Cereals (e.g., rice, oat, millet, spell, sorghum)
- Pseudocereals (e.g., buckwheat, *Amaranthus*, quinoa)
- Seeds or nuts (e.g., almond, coconut, hazelnuts, walnuts, pistachios)
- Oilseed plants (e.g., hemp, flax, sesame, sunflower)
- Vegetables (e.g., potatoes).

The most popular cow milk substitutes are soy, almond, coconut, oat, and rice beverages [16]. Cereal species, such as oats, barley, or buckwheat, are widely used in the food industry due to their valuable chemical composition [15,16,17,18,19,20]. Cereal-based beverages are available in different flavors, may or may not contain added sugars, can be low-fat and supplemented with vitamins and minerals, and are also environmental-friendly.

Cereals and pseudocereals are used as ingredients in many native fermented foods and beverages produced from legumes, fruits, and vegetables (such as soy sauce, pickles, or silage) or using lactic acid bacteria (LAB), yeast, and/or fungi (such as beer, bouza, boza, or sake) [21,22,23,24,25,26]. Many traditional beverages are made on the basis of cereals or pseudocereals. Boza, for example, is obtained through the fermentation of barley, oat, rye, millet, maize, wheat, or rice [21,26,27]. Not all the traditional cereal-based beverages are characterized by a yogurt-like texture. Some examples are togwa (a sweet and sour, nonalcoholic beverage, produced from the flour of maize, sorghum, and finger millet and, sometimes, cassava root), mahewu (maize or sorghum fermented with millet or sorghum malt), and kvass (fermented rye bread beverage) [28,29,30].

In recent years, there has been an interest in obtaining fermented cereal- or pseudocereal-based beverages that can be used as substitutes for fermented products obtained from cow milk similar to the conventional products in terms of textural and organoleptic properties and the ability to maintain viable lactic acid bacteria and probiotics during storage time [12,31]. Therefore, this review aimed to describe the basic components of cereal- or pseudocereal-based beverages and to determine the feasibility of fermenting them with LAB to obtain products with live and active LAB cells and increased nutritional value.

## 2. Characteristics and Nutritional Value of Beverages from Cereals and Pseudocereals

**Figure 1:**
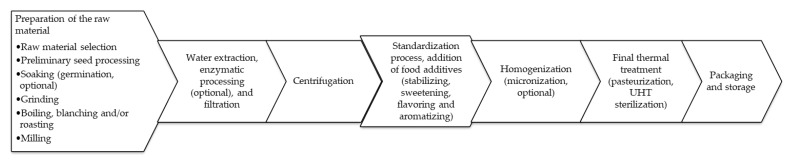
The most important processes of the production of cereal and pseudocereal beverages (details are in the text).

A plant-based beverage is an emulsion that resembles cow milk in consistency and appearance [3,32,33,34,35]. The technology used for the production of vegetable milk substitutes primarily involves the extraction of selected plant material. Depending on the type of raw material used, the technology applied for beverage production differs [13,15,35,36,37]. Cereal- and pseudocereal-based beverages are obtained using seeds or seeds without cover. The production of all plant beverages is based on a similar principle (Figure 1). In general, the steps involved in production are the aqueous extraction of the plant material (previously crushed), removal of solid parts, and finally thermal treatment of the obtained fluid [3,32,33,34,35].

The first stage is the preparation of the raw material for processing. For example, if seeds are the raw material, then following the removal of contaminants, they are soaked, filtered, and flushed. In the case of cereals and pseudocereals, they are first arched, classified, and washed. During preliminary seed processing, the physical barriers are removed and the fiber content is changed to access hydrolytic enzymes and increase the speed of hydrolysis [38]. For some raw materials, prior to soaking and grinding, additional blanching and/or roasting is performed (Figure 1). Blanching is usually carried out in boiling water for 1–5 min, and its purpose is to prevent the emergence of foam on the drink during the further process. Roasting is done at temperatures above 100 °C, in hot air. Depending on the type of raw material and temperature used, the time of roasting varies. This process allows improving the taste and aroma of the final product, but can reduce protein solubility and extraction efficiency [13]. Soaking and milling prepare the raw material for further stages and facilitate the release of nutrients contained within the material therein. Water inactivates some inhibitors and reduces the amount of phytic acid, which increases the absorption of nutrients and their bioavailability [13,35,36,37,39].

The prepared seeds or grains of cereals and pseudocereals are subjected to extraction and filtration (Figure 1) [40,41]. Filtration, centrifugation, and homogenization result in the final beverage characterized by stable consistency. For cereal beverages, processes such as enzymatic treatment, decanting, and homogenization are applied further [15,35,40,41]. Enzymatic processing (swinging, germination, addition of enzymatic preparations) is carried out, among other processes, to enable the distribution of oligosaccharides and non-starch polysaccharides that are responsible for the ideal consistency of beverages, as well as starch distribution [36,42,43,44,45,46,47,48,49]. In the case of some plant raw materials, enzymes are also added at this stage, to activate the enzymatic hydrolysis of starch or other polysaccharides. One such enzyme is alpha-amylase, which catalyzes the hydrolysis of α-1,4-glycoside amylose and amylopectin in starch, and produces compounds with shorter chains, mainly dextrins. Proteolytic enzymes that can enhance the protein solubility and efficacy of extraction and improve the stability of water suspension are also used [13,50]. Alternatively, dry raw materials are pre-ground and then subjected to aqueous extraction at an elevated temperature [13,35]. During contact with water, the layout is additionally heated to induce starch hydrolysis. Starch gelatinization increases the viscosity of plant ingredients before fermentation and also prevents phase separation [51,52]. At this stage, the above-mentioned enzymes may be added to induce hydrolysis [13,39]. It should be noted that enzymatic treatment can modify the remaining noncarbohydrate ingredients of plant beverages. Some researchers have reported that the germination of legume seeds can influence the level of B vitamins [53,54]. Ziarno et al. [54] showed that the germination process modified the fatty acid profile of bean lipids. It can be assumed that a similar phenomenon may occur during the germination and swarming of the seeds of cereals and pseudocereals. Moreover, thermal treatment at high temperatures can change the positional distribution of fatty acids in plant triacylglycerol lipids [55].

The obtained plant-based beverage base is subjected to a standardization process to obtain a product with a previously assembled composition. The pH of the final beverage is determined, and appropriate buffering substances (e.g., phosphates) are used for adjusting the values [35,40]. To increase the stability of the product before the final thermal course, hydrocolloids of plant origin are added (Figure 1). Sometimes, the obtained suspension is homogenized and micronized to increase the physical stability of the system without the need for the addition of hydrocolloids. The micronization process consists of the simultaneous crushing and mixing of the dispersed phase particles, while the liquid heterogeneous system is processed by a high-pressure homogenization gap (15–25 MPa). The size of the particles in a micronized drink usually ranges from 0.5 to 10 μm, due to which the drink is characterized by greater homogenization compared to the beverage before micronization [13,35,50].

The prepared drink is treated thermally (in a pasteurized or sterilized ultra-high temperature system), and then spilled into unit packaging (Figure 1) [13,35,36,40,41,50].

Plant-based beverages differ in their composition and nutritional value, including the content of proteins, lipids, and carbohydrates, and glycemic index [5,35]. An additional advantage of these beverages is that they are rich in digestive fiber. Furthermore, plant-based beverages do not contain lactose or cholesterol, which are present in mammalian milk [35]. The nutritional properties of plant-based milk substitutes are determined by the used plant source, processing, and fortification. Plant-based beverages may contain oil, sweetening substances, and salt, and may be enriched with calcium and vitamins (e.g., A, D) [5,13,36]. Enriched cereal- and pseudocereal-based beverages can be included in the human diet as an excellent source of calcium and vitamins, including of the A, D, and B group [13,36]. However, it is important to ensure that the fortifying substances used in these beverages are highly bioavailable and stable and do not cause excessive changes in the quality of the final product [9,12,13,50,56,57]. The final chemical composition of plant-based beverages also determines the fermentation capability of LAB.

### 2.1. Rice and Rice Beverages

There are about 20 known species of rice, of which the most popular is *Oryza saliva* L., which occurs in two subspecies: Japonica rice (also called sinica rice; abundant in Japan, Korea, and China) and Indica rice (common in most other regions of Asia). Rice grains are rich in carbohydrates (Table 1). Starch is the major carbohydrate in rice accounting for 90% of total carbohydrates, and in some species, adequate proportions of amylose and amylopectin are responsible for the starch structure and its absorption. Some amounts of fat (0.7–2%), proteins (6–7%), and ash (0.7–1.2%) are also present in rice (Table 1) [5,16,35,58,59]. The content of protein is very low, and the amino acid that limits the use of rice protein by the human body is threonine. Rice also contains minerals (phosphorus, potassium, magnesium, selenium) and vitamins (niacin, folic acid salts, choline, and vitamin E) [59]. Among fatty acids, monounsaturated acids (mainly C18:1) are dominant, while some amounts of saturated (C16:0) and polyunsaturated (C18:2) fatty acids are also present [59].

Rice-based beverages, with a whitish color, are often produced from brown rice and water along with vegetable oils (Table 1). Based on a comparison of the content of nutrients in rice and boiled rice, it can be concluded that the technological process used for rice production causes a reduction in its nutritional value [60]. Rice beverages commercially available in the market mainly consist of water and carbohydrates (including starch, glucose, and maltose), as well as traces of lipids and proteins [5,7,60]. Furthermore, these are not a good source of vitamins and microelements [61,62,63]. Therefore, commercial rice-based beverages are often enriched with B vitamins (e.g., B3 and B12), iron, calcium, and lipid components (e.g., derived from safflower or sunflower oil). Although these beverages belong to the group of plant beverages with a low nutritional value and a high glycemic index, they are consumed due to their highly hypoallergenic nature [5,7,16].

### 2.2. Oat and Oat Beverages

*Avena sativa* L. is the most popular among oat species. The basic chemical constituent of oat grains (Table 2) is carbohydrates, the proportion of which accounts for approximately 50–60% in husked oat [5,16,35,58,59,67]. Oat only contains about 1% mono- and oligosaccharides, including sucrose, raffinose, fructose, and glucose, while the leading polysaccharide is starch [59,68,69]. Compared to other cereals, oat has the highest amount of digestive fiber. However, the fiber fraction of oat is extremely valuable due to the high content of beta-glucans (non-starch polysaccharides) [9,64,68,70]. Due to the presence of exogenous amino acids, namely threonine, methionine, lysine, phenylalanine, tyrosine, valine, and leucine, oat grains are considered a valuable protein source with a high nutritional value [71]. The content of lipids in oat accounts for approximately 4–7%, with palmitic, oleic, and linoleic acids found to be dominant based on the climatic conditions, soil type, and characteristics of the plants [68,71]. Husked oat is rich in minerals, especially calcium, magnesium, phosphorus, potassium, iron, or silicon, but poor in sodium. Oat grains contain several vitamins, but only thiamine, pantothenic acid, and vitamin E are found in adequate amounts. Oat is also a source of bioactive compounds characterized by antioxidant activity, including phytosterols, polyphenols, and phytic acid salts [9,68,69,70,72].

Oat-based beverages are preferred by consumers due to their delicate and sweet aftertaste, although they do not contain significant amounts of sucrose, fructose, or glucose, but only starch and maltose [5,7,72]. Oat-based beverages available in the market contain fats and proteins, the content of which is determined by technological parameters and the needs of consumers, while experimental oat-based beverages used for obtaining fermented products may contain higher levels of carbohydrates, proteins, and/or lipids [5,16,35,58,59].

### 2.3. Millet and Millet Beverages

*Panicum miliaceum* L. is a grain crop with various common names, including millet or proso millet [73]. It is one of the oldest cereals known to have been consumed by humans and probably the first cereal used in bread production. Millet is rich in carbohydrates, mainly starch, and contains a small fraction of fiber (Table 3) [35,63,65,73]. In addition to starch, millet groats contain other carbohydrates, such as sucrose, glucose, fructose, stachyose, and raffinose [74,75,76,77,78]. Millet groats are rich in exogenous amino acids, including leucine, isoleucine, and methionine [63]. Although millet only has a small amount of fats [63,74], it has a high nutritional value as unsaturated fatty acids account for as much as 83% of total fat content, with linolenic acid playing a key role. Millet groats also contain B vitamins, such as thiamine, riboflavin, niacin, pantothenic acid, and pyridoxine, and have twofold more vitamins B1 and B2 compared to rye or wheat [65,79]. The amount of mineral salts is also higher compared to wheat, rye, or barley [59]. Millet groats have a high content of potassium, a similar content of calcium and phosphorus as wheat grains, maize, or sorghum, and a significantly higher amount of iron [65,79]. Millet is also rich in antioxidants, such as phenolic acids (e.g., ferulic acid, coumaric acid, chlorogenic acid) and flavonoids.

Market millet beverages, produced from whole grains, and made of cereal, flakes, or flour, often contain sunflower oil and salt [13,39,65,79]. If full grains are used for production, they are properly washed, soaked for at least 12 h, and then germinated and dried. If millet groats are used, the raw material is carefully rinsed to eliminate the bitter aftertaste [13,39]. Unfortunately, the scientific literature presents only limited data on market millet beverages, and the exact characteristics of these products are thus unclear. However, based on the chemical composition of raw materials used to prepare milling beverages and the data obtained on experimental beverages, it can be concluded that market millet beverages may contain an 8- to 10-fold lower content of carbohydrates, proteins, and lipids, which can be attributed to the aqueous extraction of the raw materials used [35,59].

### 2.4. Sorghum and Sorghum Beverages

*Sorghum bicolor* (L.) Moench is a typical sorghum species [65,78]. This is a drought-tolerant plant belonging to a secular family. The main ingredient of sorghum grains is starch (Table 4) [58,59,64,65,67]. Sucrose, glucose, galactose, fructose, mannose, xylose, stachyose, raffinose, fructans, and celluloses are some of the straight-chain and complex carbohydrates found in raw sorghum grains [75,76,77,78,80,81]. Sorghum is also a source of protein and lipids [59], and contains B vitamins (especially niacin), as well as macro- and microelements (such as phosphorus, potassium, selenium, and magnesium) [59]. Sorghum seeds are rich in polyunsaturated fatty acids, particularly C18:2 linoleic acid, and monounsaturated fatty acids, including C18:1 acids [59]. A characteristic property of sorghum is the presence of polyphenols (e.g., 3-deoxicinin) [59].

Unfortunately, data regarding market or experimental beverages obtained from sorghum are limited in the scientific literature, and therefore, the nutritional value of these beverages cannot be accurately determined. However, as in the case of other cereal or pseudocereal beverages, it can be assumed that the basic content of nutrients in the raw materials will be reduced by approximately 8- to 10-fold at the stage of the aqueous extract preparation.

### 2.5. Buckwheat and Buckwheat Beverages

Buckwheat (*Fagopyrum esculentum* Moench) is widely cultivated across the world due to its beneficial effects on our body [15,82,83,84,85]. Buckwheat and its products are rich in various nutrients (Table 5). The basic component of buckwheat grain is starch, the content of which depends on variety and cultivation conditions [15,82,85]. Buckwheat has a well-balanced composition of amino acids and hence is considered as most advantageous compared to other cereals [59,82,86,87]. It also has a small amount of lipids (most in the embryo, and least in the hull; oleic and linoleic acids are dominant fatty acids), vitamins (thiamine, riboflavin, niacin, pantothenic acid, pyridoxine, folic acid, and vitamin E), minerals (zinc, copper, iron, phosphorus, potassium, magnesium, selenium), digestive fiber, and valuable flavonoids, such as rutin, quercetin, orientin, vitexin, isovitexin, and iso-orientin [59,82,86,88,89,90,91].

Buckwheat beverages are obtained from whole buckwheat seeds, groats, or flakes [15,85]. As the scientific literature lacks data on the basic chemical composition or nutritional value of market millet beverages, it can be concluded based on the available data on experimental buckwheat beverages that these products contain 4.69% of carbohydrates (including 0.16% of sugars), 0.75% of proteins, and 0.16% of fats (including 0.04% of saturated fatty acids) [15,85]. The processing of raw buckwheat seeds causes changes in the carbohydrate present in them. However, the information provided by the literature is contradictory and unclear. Phiarais et al. [92] and Campbell [93] reported that sucrose is the dominant carbohydrate in buckwheat, while xylose, glucose, arabinose, and melibiose are found in smaller amounts. Kowalska and Ziarno [85] stated that buckwheat contains seven carbohydrates, namely xylose, melibiose, fructose, arabinose, glucose, sucrose, and maltose. Another study demonstrated that the glucose content in buckwheat seeds increased with an increase in the amount of water and heating time [94].

### 2.6. Amaranthus and Amaranth Beverages

The seeds of many species of *Amaranthus* are considered edible, but only *Amaranthus caudatus* L. and *Amaranthus hybridus* are used for consumption [95]. Amaranth seeds are rich in starch (Table 6), while simple sugars constitute less than 1.7% (mainly sucrose and glucose, and lesser amounts of maltose and fructose) [59]. Due to their high protein content, amaranth seeds are included in vegetarian diets [59]. Minerals found in amaranth seeds are phosphorus, potassium, magnesium, zinc, manganese, and selenium. *Amaranthus* seeds also contain several antioxidants, including rutin, isoquercetin, lectin, amaranthine, and agglutinin [96]. They also have a smaller amount of vitamins; however, the content of tocopherols deserves a special mention, as well as lutein and zeaxanthin. Among fatty acids, polyunsaturated fatty acids are the dominant lipids in amaranth seeds [59].

As the scientific literature lacks data regarding market or experimental amaranth beverages, it can be concluded that the content of chemical constituents of raw materials will be reduced several times during aqueous extract production, as in the case of other cereal or pseudocereal beverages.

### 2.7. Quinoa and Quinoa Beverages

Quinoa (*Chenopodium quinoa* Willd.), also called Peruvian rice, is not a grain, but a pseudocereal, like buckwheat. Quinoa grains are rich in starch and digestive fiber (Table 7) [5,16,59]. They also have a high amount of proteins (similar in composition to animal proteins), vitamins (thiamine, riboflavin, niacin, folic acid, pantothenic acid, tocopherols, and carotenoids), minerals (mainly calcium, potassium, phosphorus, manganese, selenium, copper, and zinc), and bioactive substances from the flavonoid group, which exhibits antioxidant properties. In addition, quinoa contains a low amount of lipids (about 5.5%), of which polyunsaturated fatty acids are dominant [59].

Due to the absence of gluten, a favorable nutrient profile, and the presence of bioactive compounds, quinoa is ideal for the production of pseudocereal-based beverages [5,97]. Quinoa beverages contain glucose, fructose, and maltose and starch [5,98,99,100]. As mentioned for other cereal or pseudocereal beverages, the basic constituents of raw materials used for the production of quinoa-based beverages will be lowered by approximately 8- to 10-fold at the processing stage [5,16,59].

## 3. Occurrence of LAB in Cereals and Pseudocereals and Their Fermentation Abilities

### 3.1. Occurrence and Activity of LAB

LAB occur naturally in various environments, including the surface of growing and decaying plant materials. This obviously indicates that LAB can adapt to a specific environment. This property of environmental adaptation of LAB can be related to their ability to use available nutrients by lactic acid fermentation, to tolerate and survive in different environmental conditions, and to produce antimicrobial compounds that can inhibit competing microorganisms [2,3,65,102,103,104,105].

Lactic acid fermentation is defined as the process by which energy-rich organic substances are enzymatically decomposed into simple compounds that are poorer in energy. This process, which takes place under microaerophilic or relatively anaerobic conditions, is carried out by various bacterial species that can convert sugars into lactic acid and other metabolites. Fermented products have been part of the human diet since the beginning of human civilization, which indicates that they were believed to have a positive effect on health [2,3,64,106,107,108,109,110,111,112,113,114,115,116,117,118]. Lactic acid fermentation is of two types: (1) spontaneous fermentation and (2) fermentation with the use of selected starter cultures. Of these, the latter allows for greater control of the process [33,119,120].

Both spontaneous and controlled lactic acid fermentation are applied in the food industry, including the dairy industry for producing fermented milk drinks, cheese, and butter; the meat industry for producing raw-ripening cured meats; the fruit and vegetable industry for producing vegetable silage and fermented food spices; and the feed industry for producing silage [23,119,121,122,123,124,125,126,127]. In general, products resulting from lactic acid fermentation are characterized by a desirable taste, improved digestibility, and increased bioavailability of nutrients (Table 8) [2,3,33,34,72,128,129,130,131,132]. The characteristic taste of fermented plant-based products can be related to their slight but significant proteolytic and lipolytic activity, as was demonstrated for fermented soy beverages [133]. In addition, fermentation has been shown to contribute to the formation of functional compounds such as B vitamins and antioxidants, and scientists have proven that fermented products are valuable for the prevention of diabetes and obesity [134,135,136,137,138,139,140,141,142,143,144]. In the case of plant-based raw materials, fermentation allows for the elimination of plant flavors and changes the content of phytic acid, polyphenols, and tannins [3,33,34,52,72,135,145,146,147,148,149,150,151,152,153,154].

Currently, cereals and pseudocereals are considered potential raw materials for the production of plant-based nondairy fermented beverages. For experimental and industrial purposes, starter cultures with a known composition are used, which allows for the repeatability of the process [32,33,119,120]. The fermentation of cereal- and pseudocereal-based beverages is mostly carried out with the following LAB: *Lactobacillus delbrueckii*, *Lactobacillus acidophilus*, *L. plantarum*, *Lactobacillus gasseri*, *Lactobacillus johnsonii*, *Lactobacillus paracasei*, *L. casei*, *Lactobacillus rhamnosus* (now classified as *Lacticaseibacillus rhamnosus*), *Lactobacillus fermentum* (now classified as *Limosilactobacillus fermentum*), *Lactobacillus reuteri* (now classified as *Limosilactobacillus reuteri*), *Lactobacillus helveticus*, *Lactobacillus lactis*, *Leuconostoc* sp. (*L. lactis* subsp. *cremoris, L. lactis* subsp. *lactis*), *Lactococcus* sp. (*L. cremoris*, *L. diacetylactis*, *L. intermedius*), and *Streptococcus thermophilus* (Table 8). Most of these bacteria have been acknowledged as “generally recognized as safe”, which suggests that they pose no risk to the health of humans after consumption. Consuming LAB at an amount of 10^9^ cells/day can have beneficial effects on health [10,99,101,118,131,155,156,157,158,159,160,161,162].

One of the issues studied is the production of fermented cereal- or pseudocereal-based beverages without the addition of thickeners or stabilizers. For this purpose, LAB producing exopolysaccharides (EPS) are studied (Table 8) [11,100,163,164,165,166,167]. EPS-synthesis is a strain-dependent metabolic characteristic, affected by the composition of the matrix and fermentation settings [168,169]. LAB can produce different types of EPS through the linking of different monosaccharides (mainly glucose, rhamnose, or galactose in the case of heteropolysaccharides) or the same polymeric unit (mainly glucose or fructose in the case of homopolysaccharides). The synthesis of EPS is correlated to LAB sugar metabolism, linking the anabolic pathway of EPS production, and the catabolic pathway of glycolysis [170]. The synthesis of EPS during the fermentation of cereal or pseudocereal beverages by lactic acid bacteria is crucial for obtaining a final product with proper texture. The advantages of EPS production during fermentation are not limited only to textural properties—they also include the enhancement of mouth-feel properties and water-holding properties [11,164,171].

Fermented plant-based beverages are often enriched with prebiotic oligofructose and inulin, which stimulate the growth of LAB [2,3,34,155,172,173,174]. Thus, some cereal- or pseudocereal-based beverages are advantageous over others due to the natural content of prebiotic substances, which in the case of cereal products include water-soluble fiber (e.g., β-glucan), oligosaccharides (galacto- and fructooligosaccharides), and resistant starch [3,33].

### 3.2. Changes in Carbohydrates Content

Beverages made from cereals or pseudocereals are an excellent matrix for the growth of LAB. As can be seen in the above discussion, the largest percentage of carbohydrates in cereals, pseudocereals, and their preparations used in the production of plant-based beverages is starch (Table 8). The process of amylolytic starch hydrolysis by enzymatic treatment, malting, or sprouting allows for the partial decomposition of starch and the release of sugars that are more easily fermented by LAB [15,85,175,176]. Starch is a plant polysaccharide formed by the condensation of D-glucose molecules linked by α-glycosidic bonds. It is not chemically homogeneous, and its structure can be divided into two fractions: amylose (essentially unbranched) and amylopectin (branched). The difference in the structure of individual starch fractions is related to the bonds linking the glucose molecules and the plant species. Amylose has only α-1,4-glycosidic bonds, while amylopectin also has α-1,6-glycosidic bonds, which enable branching [177]. During the germination of seeds, α- and β-amylases are released, which partially hydrolyze the α-1,4-glycosidic bonds of starch (but also glycogen), giving rise to maltose [15,85].

Furthermore, carbohydrates are formed as a result of starch hydrolysis, during the lactic acid fermentation of cereal and pseudocereal beverages. The content and type of carbohydrates formed depends on the cereal or pseudocereal used, the amount of water added, the thermal treatment applied during beverage preparation before fermentation, and the bacteria used for the fermentation process and process parameters [15,85,99,100]. However, the differences are mainly attributed to variations in the fermentation ability of LAB, resulting from their different biochemical activities (mainly saccharolytic activity and fermentation) [15,85,92,178].

LAB use carbohydrates as their major carbon source [178,179,180,181]. Glucose is the main energy source for living microorganisms, although some LAB also prefer fructose or lactose [181,182,183]. Glucose is also the primary carbohydrate used as a carbon source in the lactic acid fermentation process. It is a monosaccharide belonging to the group of aldohexoses, contains six carbon atoms, and is commonly found in nature. In turn, fructose is a monosaccharide belonging to the group of ketoses. It is identical in chemical formula to glucose but differs in structure. Fructose and glucose are components of the disaccharide sucrose (both linked by an α-1,4-glycosidic bond). Starch is known to be hydrolyzed by both lactic streptococci and lactobacilli. For example, Minerva et al. [179] reported that an acidophilic enzyme secreted from the cells of the strains from *Lactobacillus plantarum* (now classified as *Lactiplantibacillus plantarum* subsp. *plantarum*) hydrolyzed soluble starch, amylopectin, and to some extent amylose, without any effect on dextran and cyclodextrins. It is also known that the fermentation of starch results in the formation of other metabolites, including short-chain fatty acids (such as acetic, butyric, and propionic acid), which differ in their concentration and distribution based on the microorganisms used and carbohydrate content [180]. However, there are no data in the literature supporting that such LAB are used in industries for the production of plant-based beverages. It can be assumed that the biochemical activity of LAB will cause further changes in the carbohydrate content when the fermented cereal- or pseudocereal-based beverages are refrigerated for storage [15,85].

In general, during fermentation, the levels of carbohydrates and some indigestible poly- and oligosaccharides reduce in cereals and pseudocereals (Table 8). The raffinose group of oligosaccharides (RFO), which includes raffinose, stachyose, and verbascose, is an interesting group of oligosaccharides found in plant material, particularly grains and seeds. These oligosaccharides consist of two or more simple sugars linked together [184,185,186]. Raffinose is a trisaccharide with glucose, fructose, and galactose; stachyose is a tetrasaccharide composed of two galactose molecules, one fructose, and one glucose molecule; and verbascose is a pentasaccharide made up of four galactose and one fructose molecule. Several studies have confirmed the ability of LAB to ferment the oligosaccharides available in the plant matrix [36,178,187,188]. It has also been shown that LAB strains exhibit a high activity of enzymes such as α- and β-galactosidases [189,190,191]. Mital and Steinkraus [184] identified that α-galactosidase in lactobacilli is active at a pH of 4.5–8.0. The enzymatic activity often correlates with the catabolism of α-galactosidase, which is a characteristic of strains from *L. plantarum* and *Lactobacillus casei* subsp. *casei* (now classified as *Lacticaseibacillus casei* subsp. *casei*), while β-galactosidase activity is high in strains from species *L. plantarum* and *Leuconostoc mesenteroides*. Strains of *L. plantarum* and *L. casei* subsp. *casei* have been characterized with moderate-to-high galactosidase activity. Galactosugars are compounds that are resistant to the activity of enzymes in the digestive tract, but are used by microorganisms, including lactobacilli, during the process of lactic acid fermentation [135,186]. The above-mentioned strains have also been shown to hydrolyze RFO [184,185,186,187,188,189,190,191,192]. In fermented beans, the content of complex carbohydrates (stachyose, raffinose, verbascose) was found to be changed, but the degree of their reduction was determined by the type of microorganisms used in the fermentation process [36,43,152,153,193,194]. On the other hand, Granito et al. [193] demonstrated that, in addition to the bacteria used for fermentation, the parameters of the lactic acid fermentation process played a key role. The enzymatic degradation of stachyose and raffinose results in the formation of sucrose, fructose, and glucose, along with a change in the sweetness of the drink, its flavor, its profile of phenolics and flavonoids, and its antioxidant capacity. Similar effects can be expected in the case of fermented cereal and pseudocereal beverages, although there are no data in the literature regarding this subject.

### 3.3. Changes in the LAB Population

The number of live LAB is an important indicator of the quality of fermented beverages. A microbial cell count of 7–8 log CFU/mL indicates that the product has probiotic properties [15,85,195]. The primary criterion that ensures the health quality of the products is the viability of microorganisms from the starter culture (Table 8). Thus, the appropriate selection of starter cultures and storage parameters is essential to achieve final products with good organoleptic properties, which are determined by the metabolites formed during the fermentation process as well as during storage [15,85,131,157,196,197,198]. The effective growth of LAB during the fermentation of cereal- or pseudocereal-based beverages is dependent on the presence of significant amounts of mono- and disaccharides in the plant matrix.

Ziarno and Zaręba [131] investigated the viability of yogurt bacteria in rice-based beverages. The authors tested seven commercial freeze-dried yogurt starter cultures and noticed that the survival rate of lactobacilli was worse than streptococci, which may be due to the negative influence of antimicrobial substances derived from the plant matrix, low pH, and inappropriate refrigeration storage conditions [15,85,131,148,196,199,200,201,202,203]. As Němečková et al. [204] indicated, fermented plant-based beverages have a lower content of buffering substances compared to milk fermented with LAB, which is also reflected by the different dynamics of fermentation and the final pH values. Furthermore, the reduction in the number of bacterial cells during cold storage may have been caused by the production of antimicrobial compounds (e.g., hydrogen peroxide, bacteriocins, organic acids) by bacteria [2,15,65,85,99]. Although, the growth and viability of LAB are limited, at the same time this protects the final product against over-acidification during the distribution and refrigerated storage.

Using the *L. rhamnosus* GG strain, Kocková et al. [2] conducted an analysis on various parameters of fermentation such as pH, the number of bacterial cells, and the concentration of organic acids formed before and after 10 h of fermentation of 10 aqueous extracts obtained from a variety of cereals and pseudocereals (rye flour, rye grain, barley flour, whole grain barley flour, amaranth flour, amaranth grain, buckwheat flour, whole grain buckwheat flour, oat flour, millet grain). The authors noted that the studied strain grew in each of the tested cereal and pseudocereal substrates during the lactic acid fermentation process. In addition, the active metabolism and growth of LAB cells was observed from an initial value of 5.0–6.5 log CFU/g to a final value of 7.4–8.8 log CFU/g. During lactic acid fermentation, *L. rhamnosus* GG produced organic acids (lactic, acetic, and citric), causing a reduction in the pH value from 4.9–6.1 (initial) to 4.3–5.9 (final) [2]. In turn, during storage at 5 °C for 21 days, the population of *L. rhamnosus* GG and the pH value were found to be reduced (due to an increase in the concentration of lactic, acetic, and citric acids) [2]. In particular, a visible decrease in the *L. rhamnosus* GG population was observed in the samples obtained from buckwheat, rye, barley, and amaranth flours. Němečková et al. [204] also highlighted the negative effect of pH on the LAB population. The authors fermented beverages made from rice, rice, barley, and maize flours, supplemented with glucose (1%, w/w), to increase the content of fermentable carbohydrates. They used different LAB starters, including those from *L. delbrueckii*, *L. fermentum*, *L. casei* subsp. *casei*, *L. paracasei* subsp. *paracasei*, *L. helveticus*, *L. gasseri*, *Lactococcus lactis* subsp. *lactis, L. lactis* subsp. *cremoris, L. lactis* subsp. *lactis* biovar *diacetylactis*, and *L. mesenteroides*. Lactic acid fermentation was carried out at 37 °C (culture with lactobacilli) or 30 °C (culture with mesophilic bacteria). The course of lactic acid fermentation and the final pH of the fermented beverages (after 16 h of fermentation, pH of 3.7–4.5) depended on the LAB cultures used, while the final number of microbial cells was estimated at 7–8 log CFU/mL [195,204]. Similar observations were made by Ziarno et al. [205], who fermented millet-based beverages using a starter containing typical yogurt microflora (two species of LAB: *L. delbrueckii* subsp. *bulgaricus* and *S. thermophilus*). The authors found that the fermented drink had more than 6 log CFU/mL viable LAB cells after 28 days of storage at 6 °C.

The fermentation and biochemical activity of LAB cells, which are specific for type, species, and even strain, also translate into changes observed in the pH of fermented cereal- and pseudocereal-based beverages during cold storage (Table 8). Kowalska and Ziarno [85] reported that the following commercial yogurt starter cultures carried out the effective fermentation of buckwheat-based beverages for up to 5 h: ABY-3 (containing *S. thermophilus*, *L. delbrueckii* subsp. *bulgaricus*, *L. acidophilus* La-5, and *Bifidobacterium animalis* subsp. *lactis* BB-12), YO-MIX 207 (containing *S. thermophilus*, *L. delbrueckii* subsp. *bulgaricus*, *L. acidophilus*, and *Bifidobacterium lactis*), YO-MIX 205 (containing *S. thermophilus, L. delbrueckii* subsp. *bulgaricus, L. acidophilus*, and *B. lactis*), and VEGE 033 (containing *S. thermophilus, L. delbrueckii* subsp. *bulgaricus, L. acidophilus* NCFM, and *B. lactis* HN019). The lactic acid fermentation by each of these industrial cultures stabilized the final pH at a value below 5.0. Similar pH values were observed for a soybean beverage obtained after lactic acid fermentation [36,158]. On the contrary, Rathore et al. [206] showed that barley malt fermented with *L. plantarum* NCIMB 8826 and *L. acidophilus* NCIMB 8821 strains at 30 °C had a pH value of about 4.0. These differences in results may be related to the specificity of plant matrices, as well as the different bacterial cultures used in the studies.

## 4. The Importance of LAB for Properties of Cereal- and Pseudocereal-Based Beverages

### 4.1. Lipid Transformation

The biochemical activity of LAB is not just limited to carbohydrate fermentation. It is known that these bacteria have an intracellular system of hydrolytic enzymes, especially lipases and esterases, which catalyze the conversion of lipids and fatty acids released as triacylglycerides (TAGs) during the production of certain dairy products, such as rennet-ripened cheese [210,211,212,213]. The esterases and lipases of LAB can hydrolyze many free fatty acid esters such as tri-, di-, and monoacylglycerols. It should be noted, however, that these are intracellular enzymes; therefore, a long maturation time and subsequent bacterial cell lysis allow these enzymes to exhibit lipolytic activity during long-term maturation, which is observed in the production of ripened cheeses but not in the case of fermented beverages [214]. Pérez Pulido et al. [215] detected several strains that can exhibit lipolytic activity among lactobacilli, mainly heterofermentative strains of lactobacilli from *Lactobacillus brevis* (currently classified as *Levilactobacillus brevis*) and *L. fermentum*, although the observed lipolytic activity was limited to short- and medium-chain fatty acid esters. Akalin et al. [216] found that the esterified forms of linoleic acid also acted as substrates for the synthesis of conjugated linoleic acids (CLA) by the *L. acidophilus* La-5 strain in milk yogurts. Due to the metabolism of these bacteria, the content of the fatty acid isomer 18:2cis-9, trans-11 increased almost threefold in the tested products. This suggests that such activity should also be observed in cereal- and pseudocereal-based beverages. The results reported by Barampana and Simarda [217] agree with this assumption. The authors used *L. plantarum* strains to ferment beans and observed changes in the content of stearic, palmitic, oleic, linoleic, and linolenic fatty acids after 16 h of fermentation at 37 °C.

Lactic acid fermentation with lactobacilli also causes changes in the content of some fatty acids in the sn-2, sn-1, and sn-3 positions and the proportion of individual fatty acids in the sn-2 position. This is most likely due to the transesterification process carried out by these bacteria [54]. Lipases can act specifically on a particular fatty acid or more generally on a certain class of fatty acids. The positional specificity or regiospecificity of bacterial lipases is defined as the ability of these enzymes to distinguish between two outer positions (primary ester bonds, sn-1 and sn-3 positions) and the inner position (secondary ester bonds, sn-2 position) in the TAG backbone. For instance, sn-1,3-regiospecific lipases preferentially hydrolyze sn-1 and sn-3 positions before sn-2 when they hydrolyze triacylglycerols [54].

### 4.2. Contents of Vitamins

Although most LAB are auxotrophic to many vitamins, some are capable of biosynthesizing water-soluble vitamins such as B vitamins (including folic acid, B2, and B12) [208,218,219,220,221,222,223,224,225,226]. Taranto et al. [219] showed that *L. reuteri* CRL1098, isolated from sourdough, produced cobalamin, while Burgess et al. [220] genetically modified the *Lactococcus lactis* subsp. *cremoris* NZ9000 strain for riboflavin (vitamin B2) biosynthesis, although spontaneous LAB mutants are known to overproduce riboflavin [221]. Such starter strains could be used in the future to increase the content of vitamins in fermented plant-based beverages [222,223]. This is advantageous due to the fact that cereals and pseudocereals, which naturally contain various nutrients, including B vitamins (except vitamin B12), lose a significant amount of these bioactive substances during beverage processing. Lactic acid fermentation may change the content of B vitamins in cereal- or pseudocereal-based beverages, but the changes are influenced by the LAB strains capable of vitamin B biosynthesis, incubation conditions, and parameters used for the processing of plant-based materials into beverages. This has been proven for plant matrices other than cereals or pseudocereals [36,223,224,225,226,227].

### 4.3. Enzymatic Degradation of Phytates

Lactic acid fermentation may also provide optimal conditions for the enzymatic degradation of phytates present in cereal- or pseudocereal-based beverages as complexes with multivalent cations (e.g., iron, zinc, calcium, and magnesium). The enzymatic reduction of phytate complexes can even significantly increase the content and bioavailability of minerals, which has been confirmed for some types of flour- and experimental cereal-based beverages (Table 8) [22,208,228]. Microbial phytase can hydrolyze phytic acid salts during lactic acid fermentation, and low pH conditions and fermentation temperature can favor the activity of this enzyme. This was proven by Khetarpaul and Chauhan [208], who fermented pearl millet flour using *L. brevis* and *L. fermentum* cultures at 30 °C for 72 h. The authors noted a significant reduction in phytic acid as well as polyphenols (up to 83–88% and 80–91% of the initial content). This may improve not only the bioavailability of minerals but also the digestibility of proteins and carbohydrates. Nionelli et al. [72] examined the suitability of oat flakes for making functional beverages fermented with *L. plantarum* LP09. The researchers noted that fermentation increased the polyphenols’ availability and the antioxidant activity (by 25% and 70%, respectively).

### 4.4. β-Glucosidase Activities of LAB

A significant activity of LAB, related to some carbohydrates, as well as antioxidant capacity, is β-glucosidase activity [229,230]. β-D-glucosidases remove glucopyranosyl residues from the non-reducing end of β-D-glucosides by catalysing hydrolysis of the glycosidic bond [229]. Most β-glucosidases hydrolyse a broad range of substrates (i.e., phenols, polyphenols, and flavonoids). This way, the fermentations with LAB could increase the concentrations of phyto-oestrogens, bioactive isoflavones, and phenolic compounds in plant materials, leading to a significant contribution to the nutritional attributes of fermented plant food, cereal-, and pseudocereal-based beverages (Table 8) [231,232,233,234]. It is worth noting that β-glucosidase activity can release attractive flavor or fragrance compounds from the glucosylated precursors of fermented products and increases the bioavailability of health-promoting plant metabolites. Most of this type of research has been done on fermented soybean products or fermented vegetables [230,232,233,234].

### 4.5. The Digestibility of Proteins

The digestibility of proteins in cereal- or pseudocereal-based beverages can also be improved by a mechanism other than the breakdown of phytates or polyphenols (Table 8). Lactic acid fermentation of these beverages leads to changes in the levels of proteins and amino acids. The peptidase system of starter lactic acid bacteria has a major role on the liberation of free amino acids [52,235]. Furthermore, during acidification, the activation of cereal flour endogenous proteinases is observed [236]. For example, it has been shown that lactic acid fermentation increased the level of available lysine (a limiting amino acid for cereal proteins), methionine, and tryptophan in maize, millet, sorghum, and other cereals or pseudocereals [209,237]. However, Nanson and Field [209] observed that the levels of available (free) lysine, methionine, and tryptophan were dependent on the parameters of the lactic acid fermentation process when they studied the fermentation of corn flour. Similar effects and relationships can be expected for all fermented cereal- or pseudocereal-based beverages, but there are no literature data to support this hypothesis.

## 5. Conclusions

The high activity of LAB during the lactic acid fermentation process causes similar changes in the product composition to that observed in LAB-fermented milk, including the formation of organic acids, acidification of the environment, decomposition of some carbohydrates, and digestion of proteins and lipids. Thus, fermented cereal- or pseudocereal-based beverages can be an alternative to fermented milk to meet the growing demand for this type of product among consumers.

In our opinion, there is no barrier for the application of LAB on plant-derived beverages. The effect of lactic acid fermentation on the nutritional value of fermented cereal- or pseudocereal-based beverages seems to be variable, although literature data indicate that there is an improvement in the properties of these products. Limitations can be avoided by selecting the appropriate LAB cultures to the correct formula of plant beverage based on cereals or pseudocereals. Problems resulting from unattractive flavor and textural features can be solved by using taste, flavoring, and texturating additives. A barrier related to the texture or consistency of fermented cereal- or pseudocereal-based beverages can be missed by creating the right starch gels in the final products.

The results of the discussed studies highlight that the fermentation of cereal- or pseudocereal-based beverages with LAB enhances their health-promoting properties. The good viability of LAB during cold storage allows achieving therapeutic effects that can be obtained from consuming fermented milk products, such as kefir, buttermilk, or yogurt. In addition, cereal- or pseudocereal-based beverages lack the proteins, lactose, and cholesterol found in milk. Fermented cereal- or pseudocereal-based beverages are also a good source of proteins, fiber, vitamins, and minerals. Thus, these beverages can be not only a good choice of food for people with celiac disease, milk protein allergy, or lactose intolerance, but also a new, environmentally friendly alternative for the general public.

## Figures and Tables

**Table 1:** Basic ingredients of rice product per 100 g.

Product	Carbohydrate (g/100 g)		Protein (g/100 g)	Total Lipid (Fat) (g/100 g)	Ash (g/100 g)	Ref.
	Starch and Sugars	Fibre, Total Dietary				
Rice	65.0–80.0	7.8–12.5	7.0–10.8	1.2–2.5	n.d.	[35]
Rice	77.2	3.7	7.5	2.4	4.7	[63]
Rice	77.3	2.2	7.9	2.9	1.5	[64]
White rice	n.d.	n.d.	6.7	0.4	0.5	[58]
Brown rice	n.d.	n.d.	3.5	0. 9	1.2	[58]
Brown rice	69.1	3.3	7.3	2.2	1.4	[65]
Brown rice	87.2	1.1	8.4	1.6	1.4	[65]
Rice flour, white, unenriched	79.3	0.2–0.6	6.3–7.6	1.1–1.5	0.2–0.5	[59]
Rice flour, brown	75.5		6.6–7.5	3.4–4.7	1.2–1.5	[59]
Organic rice drink, natural	7.6	n.d.	0.04	0.8	0.1	[5]
Organic brown rice drink	5.7	n.d.	0.07	0.9	0.1	[5]
Rice milk	9.4–12.7	0.3–0.7	0.3–1.3	0.9–1.1	n.d.	[16]
Rice milk	9.17–10.27	0.3–1.52	0.28–1.78	0.32–0.97	0.48	[66]

n.d.—No data.

**Table 2:** Basic ingredients of oat product per 100 g.

Product	Carbohydrate (g/100 g)		Protein (g/100 g)	Total Lipid (Fat) (g/100 g)	Ash (g/100 g)	Ref.
	Starch and Sugars	Fibre, Total Dietary				
Raw oat	57.6	10.1	13.2	6.5	n.d.	[59]
Oat	31.1–51.0	7.7–19.2	9.0–19.0	3.1–6.6	n.d.	[35]
Oat	66.3	9.7	16.9	6.9	1.7	[64]
Oats	52.8	12.5	17.1	6.4	3.2	[63]
Oat flour, partially debranned	59.2	5.5–7.5	14.2–15.1	9.0–9.3	1.9–2.0	[59]
Organic oat drink	5.4	n.d.	0.7	0.4	0.2	[5]
Oat milk (fresh)	27.30–50.01	11.53–20.07	9.70–17.30	5.20–12.40	n.d.	[16]
Oat milk	2.75	0.8	0.78	0.28	0.48	[13]

n.d.—No data.

**Table 3:** Basic ingredients of millet product per 100 g.

Product	Carbohydrate (g/100 g)		Protein (g/100 g)	Total Lipid (Fat) (g/100 g)	Ash (g/100 g)	Ref.
	Starch and Sugars	Fibre, Total Dietary				
Millet, raw	67.3	8.5	11.0	4.2	3.2	[59]
Millet	58.0–82.0	3.2–11.4	9.8–17.2	1.9–4.8	n.d.	[35]
Millet	72.8	3.8	11.0	4.2	3.3	[64]
Millet	71.5	3.0	12.0	7.2	1.9	[65]
Finger millet	59.0	19.1	7.3	1.3	3.0	[63]
Finger millet, utricle	73.0–82.0	11.7–18.6	4.9–11.3	1.3–1.6	2.0–5.0	[74]
Pearl millet	60.5	7.0	14.5	5.1	2.0	[63]
Pearl millet, naked	67.0–72.0	8.5–15.3	6.9–20.9	3.1–8.8	0.3–5.1	[74]
Pearl millet	n.d.	n.d.	16.0	4.5	2.2	[58]
Proso millet	56.1	8.5	11	3.5	3.6	[63]
Proso millet, utricle	64.0–76.0	13.1	6.4–16.0	1.7–4.1	0.8–8.8	[74]
Foxtail millet	59.1	19.1	11.7	3.9	3.0	[63]
Foxtail millet, utricle	64.0–76.0	9.4	6.4–16.0	1.6–9.3	1.5–4.3	[74]
Kodo millet	72.0	37.8	8.3	1.4	3.6	[63]
Fonio, hulled	75.0	15.7–20.7	5.1–10.4	1.8–4.5	1.0–6.0	[74]
Teff, naked	73.0–77.0	8.0	7.9–12.6	2.0–2.4	2.2–2.9	[74]
Millet flour	71.6	2.6–4.6	9.6–12.2	2.2–5.3	1.1–1.4	[59]
Millet flour	78.7	5.9	12.1	3.6	n.d.	[35]
Millet flakes	80.5	3.8	8.1	3.2	n.d.	[35]
Millet groats	71.6	3.2	11.3	2.9	n.d.	[35]
Millet, cooked	22.4	1.3	3.5	1.0	0.4	[59]

n.d.—No data.

**Table 4:** Basic ingredients of sorghum product per 100 g.

Product	Carbohydrate (g/100 g)		Protein (g/100 g)	Total Lipid (Fat) (g/100 g)	Ash (g/100 g)	Ref.
	Starch and Sugars	Fibre, Total Dietary				
Sorghum grain	67.4	6.0–8.6	9.3–11.5	3.3–3.6	1.2–1.7	[59]
Sorghum	73.8	11.8	11.0	3.2	1.8	[63]
Sorghum	50	13.8	8.3	3.9	2.6	[65]
Sorghum	n.d.	n.d.	11.0	3.3	1.7	[58]
Sorghum flour, whole–grain	70.0	4.4–8.2	6.8–10.8	3.0–3.6	1.2–1.4	[59]
Sorghum flour, refined, unenriched	74.9	1.9	9.53	1.24	0.47	[59]

n.d.—No data.

**Table 5:** Basic ingredients of buckwheat product per 100 g.

Product	Carbohydrate (g/100 g)		Protein (g/100 g)	Total Lipid (Fat) (g/100 g)	Ash (g/100 g)	Ref.
	Starch and Sugars	Fibre, Total Dietary				
Buckwheat	61.5	10.0	13.2	3.4	2.1	[59]
Buckwheat flour, whole–groats	60.6	10.0	12.6	3.1	2.5	[59]
Buckwheat groats, roasted, dry	64.7	10.3	11.7	2.7	2.2	[59]
Buckwheat groats, roasted, cooked	17.2	2.7	3.4	0.6	0.4	[59]
Buckwheat beverage	4.69	n.d.	0.75	0.16	n.d.	[15]

n.d.—No data.

**Table 6:** Basic ingredients of amaranth product per 100 g.

Product	Carbohydrate (g/100 g)		Protein (g/100 g)	Total Lipid (Fat) (g/100 g)	Ash (g/100 g)	Ref.
	Starch and Sugars	Fibre, Total Dietary				
Amaranth grain, uncooked	58.5	6.3–7.4	12.6–15	6.3–8.4	2.2–3.2	[59]
Amaranth grain, cooked	16.6	1.9–2.4	3.6–4.1	1.4–1.7	0.7–0.9	[59]

**Table 7:** Basic ingredients of quinoa product per 100 g.

Product	Carbohydrate (g/100 g)		Protein (g/100 g)	Total Lipid (Fat) (g/100 g)	Ash (g/100 g)	Ref.
	Starch and Sugars	Fibre, Total Dietary				
Quinoa, uncooked	57.2	6.1–8.0	12.2–15.2	5.6–6.6	2.4–2.4	[59]
Quinoa, cooked	17.5	2.3–3.5	3.1–5.9	1.5–2.4	0.7–0.9	[59]
Quinoa drink	3.4	n.d.	0.2	2.3	0.2	[5]
Quinoa milk	n.d.	0.43	0.57	0.11	0.04	[101]

n.d.—No data.

**Table 8:** Studies employing LAB as starter cultures in fermentation of cereal- and pseudocereal-based beverages (examples).

Matrix	Culture Used	Topic of Study	Ref.
Rice	Commercial starters	Properties of yogurt-like fermented brown rice product	[12]
Rice	*L*. *plantarum*, *L*. *brevis*, *L*. *rhamnosus*	Characterizatics of yogurt-style snack	[51]
Rice	*L*. *casei*, *L*. *bulgaricus L*. *acidophilus*, *S*. *thermophilus*, *B*. *longum*,	Probiotic rice milk	[31]
Rice	*L*. *brevis*, *L*. *fermentum*, *L*. *plantarum*, *Bifidobacterium longum*	Properties of fermented rice Beverages	[160]
Rice	Commercial starter culture (*L*. *acidophilus*, *S*. *thermophilus*, *Bifidobacterium bifidum*)	Fermented rice milk	[56]
Rice	Commercial starter cultures of yogurt bacteria	Viability of starter culture bacteria	[131]
Rice	*L*. *plantarum*, *L*. *vermiforme*, *L*. *paracasei*	Fermented rice beverage	[202]
Oat	*L*. *plantarum*	Properties of oat-based beverage	[161]
Oat	*L*. *plantarum*	Properties of fermented oat-based product	[207]
Oat	*P*. *damnosus*	Properties of oat-based product, determination of EPS	[166]
Oat	*L*. *plantarum*	Properties of flavored oat drink	[132]
Oat	*L*. *plantarum*	Properties of synbiotic functional drink from oats	[174]
Oat	*L*. *plantarum, L*. *Casei, L*. *paracasei*	Properties of oat-based, yogurt-like beverage	[72]
Oat	*L*. *brevis*, *P*. *damnosus*	Properties of oat-based product	[167]
Oat	*L*. *delbrueckii* subsp. *bulgaricus*, *L*. *brevis*, *S*. *thermophilus*	Properties of oat-based, yogurt-like beverage, determination of EPS yield	[171]
Oat	*L*. *reuteri*, *L*. *acidophilus*, *Bifidobacterium bifidum*	Properties of oat-based product	[203]
Oat	Commercial yogurt culture (*S*. *thermophilus*, *L*. *delbrueckii* subsp. *bulgaricus*)	Properties of oat yogurt-type product	[57]
Millet	Commercial yogurt culture (*S*. *thermophilus*, *L*. *delbrueckii* subsp. *Bulgaricus*, *Bifidobacterium* sp.)	Properties of fermented millet beverages	[35]
Millet	Commercial yogurt culture (*S*. *thermophilus*, *L*. *delbrueckii* subsp. *bulgaricus*, *Bifidobacterium* sp.)	Properties of fermented millet beverages	[205]
Millet	*L*. *brevis*, *L*. *fermentum*	Carbohydrate content of pearl millet flour	[208]
Sorghum	*W*. *confusa, L*. *paracasei, L*. *fermentum, L*. *brevis, L*. *plantarum*	Volatile analysis of fermented cereal beverage	[24]
Buckwheat	Commercial starter culture *(S*. *thermophilus*, *L*. *delbrueckii* subsp. *bulgaricus*, *Bifidobacterium* sp.)	Fermentation of buckwheat beverages	[15]
Buckwheat	*Lb*. *rhamnosus*, *Lactococcus lactis* spp. *lactis*, *L*. *lactis* spp. *cremoris*, *S*. *thermophilus*	Growth and metabolic characteristics of selected LAB in buckwheat substrates	[195]
Buckwheat	Commercial yogurt culture *(S*. *thermophilus*, *L*. *delbrueckii* subsp. *bulgaricus*, *Bifidobacterium* sp.)	Characteristics of fermented buckwheat beverages	[85]
Quinoa	*L*. *plantarum*, *L*. *casei*, *Lactococcus lactis*	Characteristics of quinoa-based fermented beverage	[99]
Quinoa	*L*. *plantarum*, *L*. *rhamnosus*, *W*. *confusa*	Microbial, chemical, rheological, and nutritional properties of quinoa yogurt-like beverages	[100]
Quinoa	*W*. *cibaria*	Nutritional properties of quinoa-based yogurt	[169]
Quinoa	*L*. *plantarum*	Fermentation process, microbiological safety	[162]
Quinoa	Commercial starter culture *(Bifidobacterium* sp., *L*. *acidophilus*, *S*. *thermophilus)*	Nutritional properties of quinoa-based beverage fermented	[101]
Maize	*L*. *paracasei*	Properties of functional corn-based beverage	[25]
Maize	*L*. *rhamnosus*, *S*. *thermophilus*	African maize-based fermented food (kwete)	[27]
Maize	Spontaneous fermentation	Fermented cornmeal, digestibility of proteins	[209]
Emmer	*L*. *plantarum*, *L*. *confusa*, *L*. *brevis*, *W cibaria*, *P*. *pentosaceus*, *L*. *rhamnosus*	Characterization of fermented emmer, beverages	[165]
Malt, barley, and barley mixed with malt	*L*. *plantarum*, *L*. *acidophilus*	Functional and organoleptic properties of cereal-based probiotic drinks	[206]
Rice (red), barley buckwheat	*L*. *casei*, *L*. *paracasei*, *L*. *parabuchneri*, *L*. *buchneri*, *L*. *fermentum*, *L*. *coryniformis*, *L*. *rhamnosus*, *P*. *parvulus*, *W*. *oryzae*, *S*. *thermophilus*	Properties of cereal (red rice and barley)- and pseudocereal (buckwheat)-based substrates	[198]
Mixture of cereals	*L*. *rhamnosus*	Rye, barley, amaranth, buckwheat, oat	[2]
Mixture of cereals (rice, barley, emmer, oat)	*L*. *plantarum*, *L*. *rossiae*, *W*. *cibaria*, *P*. *pentosaceus*	Microbiological, textural, nutritional, and sensory properties of vegetable yogurt-like beverages	[52]
Rice, millet	Commercial starter culture *(Bifidobacterium* sp., *L*. *acidophilus*, *S*. *thermophilus)*	Bacterial population, color, flavor, texture, and overall acceptability of the beverages, shelf-life	[65]
Boza–Balkan drink (from cereals)	*L*. *plantarum*, *L*. *rhamnosus*, *L*. *pentosus*. *L*. *paracasei*	Antimicrobial activity, tolerance to gastric juice, bile salt hydrolase activity, adhesion to HT-29 and Caco-2 cell lines	[26]
